# Feature Paper in Oral Physiology and Pathology

**DOI:** 10.3390/life14070895

**Published:** 2024-07-19

**Authors:** Giuseppe Minervini

**Affiliations:** 1 Saveetha Dental College and Hospitals, Saveetha Institute of Medical and Technical Sciences, Saveetha University, Chennai 602105, Tamil Nadu, India; giuseppe.minervini@unicampania.it; 2 Multidisciplinary Department of Medical-Surgical and Odontostomatological Specialties, University of Campania “Luigi Vanvitelli”, 80121 Naples, Italy

In the realm of life sciences, the journal ‘*Life*’ has consistently served as a beacon for groundbreaking research and scientific discovery. This Special Issue continues this tradition, presenting a diverse array of studies that collectively underscore the intricate and multifaceted nature of biological research. The articles featured in this issue collectively enhance our comprehension of health and disease, with notable advancements in the realms of genetics, disease biomarkers, musculoskeletal health, therapeutic interventions, and beyond.

This Special Issue primarily concentrates on exploring the genetic and molecular pathways underlying various diseases. A notably important study examines the polymorphisms in the gene encoding heat shock factor 1 (HSF1) and their link to type 2 diabetes [1]. This research sheds light on the crucial role of protein folding and stress responses in diabetes pathogenesis, pinpointing specific genetic variations in HSF1 associated with an increased risk of the disease, especially in overweight and obese females [1,2,3]. This finding enhances our comprehension of the molecular mechanisms driving diabetes and proposes potential avenues for targeted genetic therapies.

The quest for dependable biomarkers continues to be a major research priority, especially for chronic and degenerative conditions like osteoarthritis (OA) [4]. The review of soluble and extracellular vesicle (EV)-associated biomarkers in knee OA pathology underscores the urgent need for early and precise diagnostic tools [5,6]. Scientists predict that developing non-invasive biomarkers capable of forecasting disease progression and identifying therapeutic targets will significantly enhance patient outcomes. Such biomarkers will change OA management by enabling early intervention and a more personalized approach to treatment [4,7,8,9,10,11].

This special edition also addresses the important topic of obesity and its effects on musculoskeletal health. A thorough study that looked at how cartilage works and how likely it is to break in the medial tibiofemoral compartment makes the link between having a higher body mass index and a higher risk of cartilage degradation very clear. This would imply that scientific research would more likely encourage weight control as a significant component of programs to prevent musculoskeletal diseases and how multidimensional lifestyle interventions can offset the effects on joint health [12,13,14,15,16,17,18].

Exercise functions as a crucial therapeutic intervention for mitigating chemotherapy-induced peripheral neuropathy (CIPN) [19]. Systematic reviews and meta-analyses detailed in this issue reveal that therapeutic exercise markedly alleviates peripheral neuropathy symptoms in cancer patients. These findings underscore the significance of exercise not only as an adjunctive treatment during chemotherapy but also as a strategy to enhance the overall quality of life for cancer patients [19,20]. This underscores the importance of integrating physical activity into standard cancer care protocols.

The intersection of autoimmune diseases and parasitic infections presents a unique and fascinating avenue for understanding disease mechanisms [21,22]. An intriguing study on the influence of intestinal nematode infections on growth factors in autoimmune models reveals how parasitic infections can modulate immune responses and angiogenesis. This research offers potential therapeutic insights, suggesting that manipulating parasitic infections could lead to novel treatments for autoimmune diseases [22]. This kind of finding represents the complexity of interplay among various biological systems and the possibility of cross-disciplinary ways of disease treatment.

This emphasis on emerging breakthroughs is dynamic in the development of new insights into sepsis-induced coagulopathy. Understanding the prothrombotic states induced by severe infections is crucial for developing early-stage diagnostic markers and therapeutic strategies [23,24,25]. By identifying these markers, researchers aim to improve the management and outcomes of septic conditions, which remain a significant challenge in clinical settings.

This edition also advocates for the biomedical applications of platelet-derived extracellular vesicle (PEV) transfer. PEVs now elegantly transfer a diverse array of biomolecules, modifying numerous target cell functions and supporting processes like inflammation, coagulation, and tissue repair [26,27,28]. These findings indicate promising avenues for developing novel therapeutic regimens based on PEVs for various clinical contexts. Moreover, the potential of PEVs to serve as both diagnostic biomarkers and therapeutic tools represents a major advancement in translational medicine.

Cutting-edge research on chondrocyte biology further enriches this issue. Studies on the role of WNT16 in maintaining the articular chondrocyte phenotype bring new understandings to joint homeostasis and osteoarthritis [29,30]. Studies suggest that WNT16 stimulates proliferation and sustains chondrocytes at a crucial level for cartilage homeostasis [29]. The molecular mechanism by which WNT16 works basically helps to create a number of therapeutic interventions that can stop or at least slow the progression of OA [29]. Such advancements are particularly relevant given the aging global population and the increasing prevalence of joint-related disorders.

This issue also highlights the innovative use of dupilumab for treating dermatitis associated with immunoglobulin G4-related disease (IgG4-RD) [31]. The case study in this issue showcases how targeting specific interleukins can result in significant therapeutic benefits, underscoring the potential of personalized medicine. The patient dramatically improved in quality of life after treatment with dupilumab, thus reflecting how innovative therapeutic strategies can control complex and rare diseases [31,32].

Even more suggestively, the balance between genetic predisposition and added risk from environmental factors forms a constant undercurrent in this volume. Research on genetic susceptibility in various diseases takes into account both innate genetic makeup and exogenous ecological influences [33]. This holistic approach is crucial for developing more effective and comprehensive treatment strategies that address the multifactorial nature of most diseases.

In sum, this Special Issue of ‘*Life*’ reflects the dynamic and interconnected nature of life sciences research. From genetic predispositions to innovative therapeutic interventions, the contributions within these pages highlight the relentless pursuit of knowledge aimed at understanding and ultimately improving human health. Each article, in addition to moving forward our scientific understanding, emphasizes the need for interdisciplinary collaboration in facing complex health and disease challenges. As we continue to explore new areas of biological research, the insights gained from these studies will undoubtedly aid in devising more effective strategies for improving human health and well-being.
